# Developments in the management of febrile neutropenia

**DOI:** 10.1038/bjc.2011.304

**Published:** 2011-08-23

**Authors:** J Pascoe

**Affiliations:** 1 Cancer Research UK Clinical Trials Unit, School of Cancer Sciences, University of Birmingham, Vincent Drive, Edgbaston, Birmingham, B15 2TT, UK

Febrile neutropenia (FN) is one of the most serious complications of chemotherapy. Recent advances in its management have included prevention with prophylactic antibiotics and growth factors, improvement in the management of the septic patient and the development of risk stratification systems aiming to identify those patients at high risk of complications and allowing clinicians to tailor therapy appropriately. However, despite these, FN remains the cause of significant morbidity and mortality, with healthcare resource implications (Kuderer *et al*, 2006). Research therefore continues in attempt to reduce the impact of this condition.

Two studies published in this issue have addressed the concept of risk stratification in FN, and both the articles provide information, that if developed further, may be used to inform decisions in the management of patients in the future (Carmona-Bayonas *et al*, 2011; Cheng *et al*, 2011). Historically, the standard treatment of FN has been inpatient management with intravenous antibiotics, until resolution of fever and recovery of neutrophil count. However, the knowledge that the majority of patients with FN actually have an uneventful clinical course, has led to the development of risk stratification tools in an attempt to predict those cases in which complications are likely. The most widely used instrument currently is the prospectively validated Multinational Association for Supportive Care (MASCC) index, which allows clinicians divide patients into high and low risk of complications before obtaining the neutrophil count (Klastersky *et al*, 2000). The use of MASCC index is now recommended in ESMO and other guidelines (de Naurois *et al*, 2010).

The ability to identify patients at low risk of complications has led to the development of less-intensive treatment strategies for these patients involving oral antibiotics and/or early discharge/treatment at home. Several studies (Klatersky *et al*, 2006; Innes *et al*, 2008) and a recent systematic review (Teuffel *et al*, 2011a) have demonstrated that some patients predicted to be at low risk of complications can be treated with oral antibiotics and early hospital discharge. However, studies involving risk stratification, report a proportion of patients assessed as low risk, who go on to develop complications, suggesting that although the safety of outpatient treatment is becoming established, the risk stratification of patients presenting with FN could be improved.

Carmona-Bayonas *et al* (2011) present an attempt to further refine the process. They observe that a considerable part of the MASCC criteria involves identifying those that are clearly unwell at the time of assessment. In contrast, they attempt to identify predictive factors that identify those patients that appear well at the time of diagnosis but develop complications. A pragmatic triage system is described whereby patients with FN are classified into clearly unstable and apparently stable patients (ASP), and the rate of complications in the ASP group is calculated. The MASCC score was found to have a low sensitivity for predicting complications in the ASP group, but this is not unexpected, as it is a very different patient population to that in which it was validated, which is bound to affect its sensitivity. This simply serves to remind us that caution is needed when applying risk stratification tools outside of their intended patient population. A retrospective case–control study was developed to identify risk factors for the development of complications, and multivariable analysis identified six factors that were independent predictors of complications; performance status two or greater, chonic bronchitis, chronic heart failure, stress hyperglycaemia, monocytes <200 mm^−3^ and stomatitis grade 2 or more.

This is an interesting finding and there are certainly plausible reasons why each of these findings could predispose patients to the development of complications. However, it should be remembered that this analysis was based on a relatively small number of cases that had already undergone a pragmatic risk stratification that is different to the widely adopted MASCC scoring system. Clearly these markers require validation in prospective studies if they are to become useful determinants of risk of complication and it is therefore currently unclear what role they will have in the management of FN. Perhaps consideration should be given to investigating them further, either in tandem with the MASCC criteria or applying them to patients assessed as low risk by MASCC. In this way they may have the potential to improve the sensitivity of the MASCC index and current practice.

It now appears clear that risk stratification allows identification of a proportion of patients who are at low risk of complications and that some of these patients can be managed safely as outpatients. A recent study in this journal by Teuffel *et al* (2011b) has also demonstrated, using a Monte Carlo cost–utility model, that outpatient management of low-risk FN with either oral or intravenous antibiotics was less expensive than hospital treatment. Given that outpatient treatment appears to be equally efficacious and more cost effective than inpatient treatment, it would seem obvious that patients and carers would prefer to receive treatment as an outpatient when possible. However, very little research had been conducted into patients’ preferences with regard to this. Cheng *et al* (2011) studied the effect of various treatment strategies for a hypothetical episode of FN on health-related quality of life of children undergoing chemotherapy and their carers. The different strategies were entire inpatient intravenous treatment, early discharge on oral antibiotics, entire oral outpatient treatment or entire intravenous outpatient treatment. They also determined patients and carers’ preference for the different treatment modalities. Perhaps, surprisingly, the most common preference for parents was inpatient management, whereas less surprisingly was children preferred outpatient management. Outpatient intravenous treatment/early discharge and early discharge were related to higher anticipated health-related quality of life outcomes for parents and children, respectively. The preference of parents is initially surprising; however, it is clear that the current treatment strategy in the investigating centre comprised of inpatient treatment for the duration of FN. It is, therefore, possible that parents, in particular, were concerned that outpatient management was a less safe or efficacious option and they were willing to accept the inconvenience of an admission in return for optimum care. It would be interesting to determine whether this is also the case in adults receiving treatment for FN and whether this differs according to whether patients are receiving treatment in a curative or palliative context. As there is now evidence to suggest that outpatient management may be an appropriate treatment option for some patients, further work should focus on patient and carer education to allow them to have confidence in this management strategy.

It can be seen that the use of risk stratification in FN continues to evolve and these papers provide useful and insightful information. Carmona-Bayonas *et al* (2011) provide data that may be used to further refine the process of risk stratification in order to improve the sensitivity of our assessment tools. Just as importantly Cheng *et al* (2011) emphasise the importance of educating patients and carers about developments in the management of their conditions in order that they may have confidence in new and innovative management strategies.
